# The complete mitochondrial genome of the sea spider *Nymphon gracile *(Arthropoda: Pycnogonida)

**DOI:** 10.1186/1471-2164-7-284

**Published:** 2006-11-06

**Authors:** Lars Podsiadlowski, Anke Braband

**Affiliations:** 1 Department of Animal Systematics and Evolution, Institute of Biology, Freie Universität Berlin, Konigin-Luise-Str. 1-3, D-14195 Berlin, Germany; 2 Department of Comparative Zoology, Institute of Biology, Humboldt Unversität, Phillipstr. 13, D-10115 Berlin, Germany

## Abstract

**Background:**

Mitochondrial genomes form units of genetic information replicating indepentently from nuclear genomes. Sequence data (most often from protein-coding genes) and other features (gene order, RNA secondary structure) of mitochondrial genomes are often used in phylogenetic studies of metazoan animals from population to phylum level. Pycnogonids are primarily marine arthropods, often considered closely related to chelicerates (spiders, scorpions and allies). However, due to their aberrant morphology and to controversial results from molecular studies, their phylogenetic position is still under debate.

**Results:**

This is the first report of a complete mitochondrial genome sequence from a sea spider (*Nymphon gracile*, class Pycnogonida). Gene order derives from that of other arthropods so that presumably 10 single tRNA gene translocations, a translocation of the mitochondrial control region, and one large inversion affecting protein-coding genes must have happened in the lineage leading to *Nymphon gracile*. Some of the changes in gene order seem not to be common to all pycnogonids, as those were not found in a partial mitochondrial genome of another species, *Endeis spinosa*. Four transfer RNAs of *Nymphon gracile *show derivations from the usual cloverleaf secondary structure (truncation or loss of an arm). Initial phylogenetic analyses using mitochondrial protein-coding gene sequences placed Pycnogonida as sister group to Acari. However, this is in contrast to the majority of all other studies using nuclear genes and/or morphology and was not recovered in a second analysis where two long-branching acarid species were omitted.

**Conclusion:**

Extensive gene rearrangement characterizes the mitochondrial genome of *Nymphon gracile*. At least some of the events leading to this derived gene order happened after the split of pycnogonid subtaxa. Nucleotide and amino acid frequencies show strong differences between chelicerate taxa, presumably biasing phylogenetic analyses. Thus the affinities between Pycnogonida and Acari (mites and ticks), as found in phylogenetic analyses using mitochondrial genes, may rather be due to long-branch attraction and independently derived nucleotide composition and amino acid frequency, than to a real sister group relationship.

## Background

Due to their evolutionary history as derived endosymbionts, mitochondria have retained genetic material – the mitochondrial genome. Much of their original gene content was eliminated or transferred to the nucleus [1], while only a small proportion of genes has persisted to the present. In triploblastic animals the circular mitochondrial genome is sized around 11–20 kilobases and contains typically 37 genes: 13 protein-coding genes, two ribosomal RNA genes and 22 transfer RNA genes [2]. Mitochondrial genomes serve as a simple model for modes and mechanisms of gene rearrangements and genome evolution and provide large datasets for phylogenetic analyses. The frequent use of mitochondrial genes for inferring phylogenetic relationships of animals is due to their universal distribution among taxa, strongly conserved regions in some genes (facilitating universal PCR primer sets) and the absence of paralog genes [3]. However, the incidental presence of nuclear copies of mitochondrial genes [4] and strong differences in nucleotide composition between taxa [5] may complicate phylogenetic analyses.

During the last ten years mitochondrial genome data have played an important role in redefining arthropod relationships. The position of mitochondrial *trnL2 *is changed in crustaceans and hexapods, but not in chelicerates and myriapods [6]. Also from sequence-based analyses of mitochondrial [7,8] and nuclear genes [9-11] the Pancrustacea hypothesis found strong support, while the traditional Tracheata hypothesis, mainly based on morphological data, is now widely rejected. Mitochondrial genome data also provided strong evidence towards the identification of the formerly enigmatic Pentastomida (tongue worms) as aberrant crustaceans [12]. While those hypotheses were collectively supported by nuclear and mitochondrial data, some other hypotheses obtained with mitochondrial genome data are highly disputed, as for example the polyphyly of hexapods [7,8] or the phylogenetic position of pycnogonids [13].

Pycnogonids or sea spiders are among the most bizarre arthropods, some of them with very large legs attached on a tiny body. Food uptake is performed by a pharyngeal suction tube, some species have even lost all head appendages (chelifores and pedipalps). Due to their derived morphology their phylogenetic position remains uncertain, although most workers consider them as primarily aquatic chelicerates [14]. Recent phylogenetic analyses using a combination of molecular and morphological data [11], or nuclear genes [9,10] support a basal position among chelicerates. In contrast, sequence data from partial mitochondrial genomes suggest an affinity to Acari (mites and ticks) [13], thus implying a terrestrial origin of pycnogonids. Recently, neuroanatomical data suggest that pycnognid chelifores are not positionally homologous to cheliceres [15], thus questioning pycnogonid affinities to Euchelicerata. However, hox gene expression data do not support the this view [16]. We report here the first complete mitochondrial genome sequence for a member of the Pycnogonida,*Nymphon gracile*. We use these data to analyse chelicerate relationships and to evaluate hypotheses of the phylogenetic position of Pycnogonida. We also discuss ancestral and derived features of the mitochondrial genome of *Nymphon gracile *and the influence of AT-content and differences of amino acid frequencies on phylogenetic analyses.

## Results and discussion

### Mitochondrial genome organization

The mitochondrial genome of *Nymphon gracile *is a circular DNA molecule of 14,681 bp length [GenBank:DQ666063]. All 37 genes expected for animal mitochondrial genomes have been identified. Gene overlaps (7 bp) exist between *nad4 *and *nad4L*, as well as between *atp8 *and *atp6*, as is reported for many other mitochondrial genomes. Six out of thirteen protein-coding genes show incomplete stop codons (T or TA), which is probably compensated by posttranscriptional polyadenylation [17].

**Figure 1:**
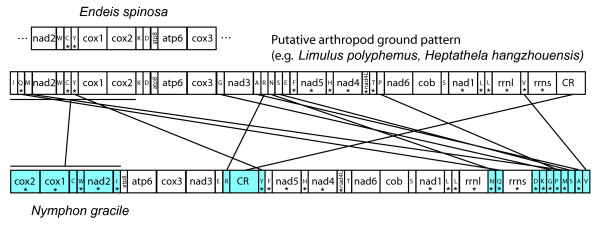
**Gene order in pycnogonids **(*Nymphon gracile*, this study; *Endeis spinosa*, according to [13]). Comparison to the ground pattern of Euarthropoda (here represented by *Limulus polyphemus *and *Heptathela hangzhouensis*). Asterisks indicate that a gene is located on the opposite strand. Transfer RNA genes are depicted by their corresponding one-letter amino acid code. Colored genes have derived relative positions in the derived gene order of *Nymphon gracile*. Lines refer to putative independent translocation events.

Gene order (Fig. 1, Tab. 1) of the mitochondrial genome differs in many positions from that of the horseshoe crab *Limulus polyphemus*, which is considered to represent the euarthropod ground pattern [18]. One large segment (about 3,500 bp) containing the protein-coding genes *cox1*, *cox2*, *nad2 *and the transfer RNA genes *trnC*, *trnW*, *trnI *were probably subject to an inversion. Maintaining their original gene order, these genes were found on the opposite strand in *Nymphon gracile *compared to other arthropods. Probably *trnK *and *trnD *were also involved in the same inversion, meanwhile separated from *cox2 *by six tRNA genes, which have different positions in other arthropods. Further translocation events of single tRNA genes may have led to their actual position in *Nymphon gracile*. Three tRNAs were lost from the inverted segment (*trnQ*, *trnM*, *trnY*) and translocated to other positions in the mitochondrial genome. Two tRNA genes (*trnE *and *trnR*) have interchanged their positions, making it impossible to decide which of them was translocated and which stayed in its position. Supposing that *trnK *and *trnD *have changed their position due to the large inversion mentioned above, altogether ten tRNA genes have undergone individual translocation events. Except from those involved in the inversion, no other protein-coding or rRNA gene has changed its position compared to the ground pattern of Euarthropoda, represented by *Limulus polyphemus *[18]. The two *trnL *genes lie adjacent to another between *nad1 *and *rrnL*, as expected for the euarthropod ground pattern. This differs from the derived condition in Hexapoda and Crustacea (*trnL2 *between *cox1 *and *cox2, trnL1 *between *nad1 *and *rrnL*).

A large non-coding region is present between *nad3-trnE-trnR *and *trnY-trnF-nad5*. This is very likely to be the mitochondrial control region, which therefore must have been translocated, too. As the strand bias of nucleotide frequencies is comparable to other arthropods (see below) the control region seems not to be inverted as it is assumed for scorpions and two web spider species [13].

A partial (5105 bp) mitochondrial genome of another species of Pycnogonida, *Endeis spinosa *[GenBank:AY731173], was published recently [5,13] and revealed no differences in gene order to *Limulus polyphemus *in the segment ranging from *nad2 *to *cox3*. Therefore we presume that the large inversion recorded in *Nymphon gracile *must have happend after the split between the two clades. Assuming a larger taxon sampling the derived gene order of *Nymphon gracile *may serve as an apomorphic character supporting a subtaxon of Pycnogonida. Six of the ten individually translocated tRNA genes of *Nymphon gracile *found their new position between *trnK *and *cox2*. In *Endeis spinosa *these two genes are adjacent, therefore these six tRNA translocations may also have happened after the split of the two clades.

### Secondary structure of RNAs

**Figure 2:**
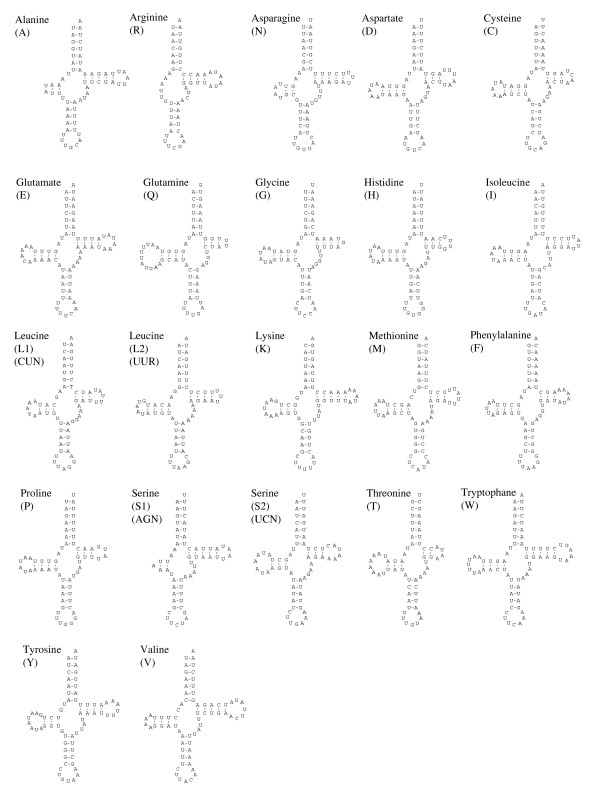
Putative secondary structures of mitochondrial tRNA molecules from *Nymphon gracile*.

Out of 22 transfer RNAs usually present in metazoan mitochondrial genomes we have identified 18 by tRNA-scan [19]. The remaining four show derivations from the typical cloverleaf structure: the DHU stem and loop is extremely short or missing in *trnA*, *trnN *and *trnS1*, while it is definitely missing in *trnR *(putative secondary structures are shown in Fig. 2). Such aberrant secondary structures are often found in metazoan mitochondrial tRNAs, some taxa even show derivations in the majority of their tRNA genes (e.g. some nematodes [20], or web spiders [21,22]). It is not clear if the function of such derived tRNAs is maintained in every case, as there are reports of recruitment of nuclear tRNAs into mitochondria [1]. In spiders however, it is very likely that tRNAs are functional, despite lacking the TΨC stem and loop [23]. *trnS1 *from the horseshoe crab *Limulus polyphemus *is also missing the complete DHU stem and loop, while all other tRNAs in this species could be folded into cloverleaf structures [24].

### Nucleotide composition

Nucleotide compositions of protein-coding and ribosomal RNA genes clearly demonstrate a strand specific bias (Tab. 2). CG-skew is positive for all genes on (+)-strand and negative for genes on (-)-strand, AT-skew is positive or only slightly negative in (+)-strand genes, while strongly negative in (-)-strand genes. This is also true for genes which have changed from one strand to the other due to an inversion during pycnogonid evolution (*cox1*, *cox2*, *nad2*). Strand bias in nucleotide composition is probably due to asymmetries during the replication of mitochondrial genomes, leading to different mutational pressures on both strands. In the literature the exact mechanisms are controversially discussed: one strand stays single-stranded during replication in the strand-displacement model [25], or is subject to extensive incorporation of ribonucleotides in the strand-coupled model [26]. Almost all arthropods show this strand bias [5,13]. In some species a reversal is seen, probably due to a strand swap of the mitochondrial control region (e.g. in scorpions and web spiders [5], as well as in an isopod [27]). As seen from the pycnogonid and spider examples, strand reversal of single genes leads to a quick reversal of strand bias, too. In performing phylogenetic analyses one has to take into account these findings and probably has to modify evolutionary models [5].

### Phylogenetic analysis

**Figure 3:**
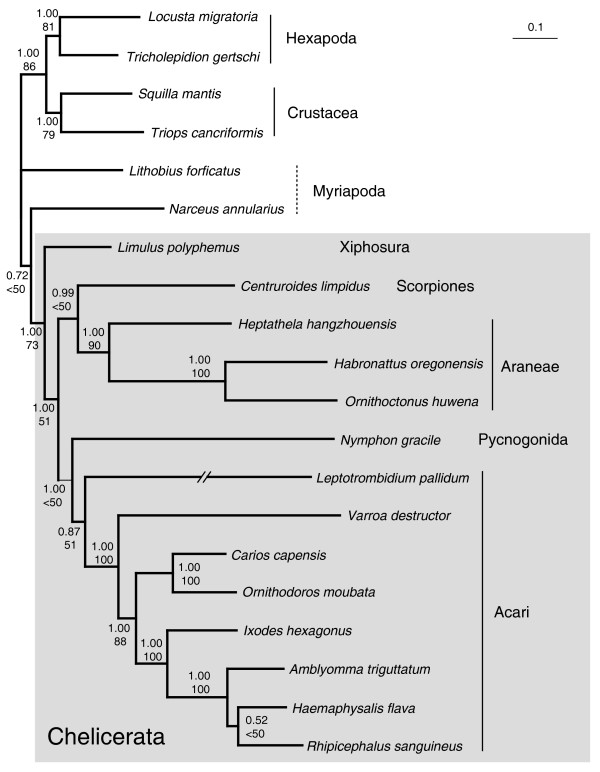
**Maximum likelihood tree of chelicerate relationships – complete dataset**. According to a nucleotide alignment (first and second codon positions) from 13 protein-coding genes. Numbers above branches indicate Bayesian posterior probabilities (upper) and bootstrap percentages of maximum likelihood analysis (lower). Branch lengths reflect substitutions per site.

Phylogenetic analysis using nucleotide sequences from all mitochondrial protein-coding genes (Fig. 3) reveals a strong support for a taxon comprised of *Nymphon gracile *and Acari (mites and ticks) from BI but not from ML bootstrap analysis. Similar results were published based on an analysis of five mitochondrial protein-coding genes [13]. This contradicts almost every other phylogenetic study which included pycnogonids (the only recent exception not based on mitochondrial genes is a combined analysis of 18S/28S sequences and morphological data, including fossils [28], where Pycnogonida, Acari and Palpigradi together form one clade – but with extreme character conflict). Recent multigene analyses place pycnogonids outside Arachnida: either (1) as sister group to Euchelicerata (Xiphosura + Arachnida), e.g. with a combined alignment of EF-1α, EF-2, and RNA-Pol II [10], as well as with a combined dataset of nine genes and morphology [11]; or (2) in an unresolved trichotomy with Euchelicerata and Myriapoda (together forming the clade Paradoxopoda or Myriochelata), e.g. using a combined aligment of 18S and 28S [9].

As a consequence of an Acari-Pycnogonid clade one would have to assume that pycnogonids may either have had terrestrial ancestors, or that an independent transition to terrestrial life was undertaken by Acari on the one hand, and spiders and scorpions on the other hand (leaving the question open where to place the remaining arachnid taxa). At least the first hypothesis is contradicted by the fossil record: the oldest pycnogonid fossil is from the upper Cambrian, a time from which no terrestrial animal is known [29] – the first terrestrial arachnids were not found before the Silurian – a gap of about 70 million years.

**Figure 4:**
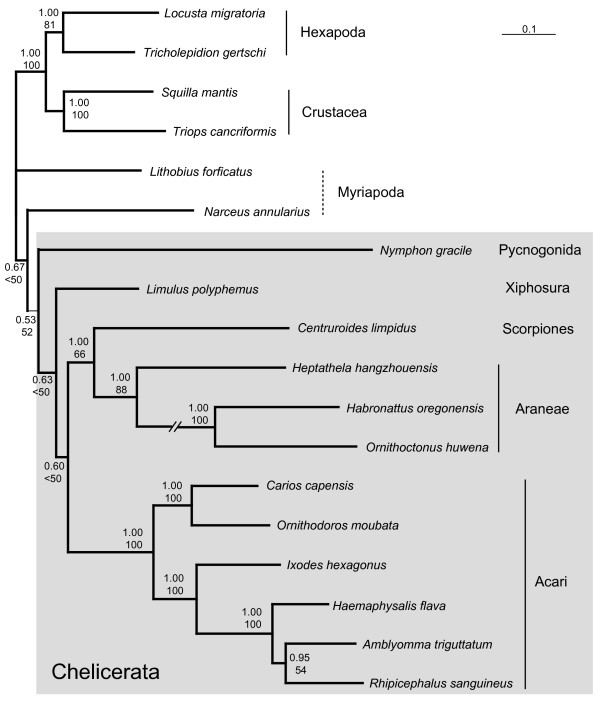
**Maximum likelihood tree of chelicerate relationships – reduced dataset**. Alignment without the long-branching acarid species *Varroa destructor *and *Leptotrombidium pallidum*. Details of analyses and legends as in Fig. 3.

To determine effects of long-branch attraction [30], a second analysis was performed, on a dataset where sequences from the long-branching acarids *Varroa destructor *and *Leptotrombidium pallidum *were omitted (Fig. 4). In the resulting tree Pycnogonida does not cluster with Acari, rather appears as sister taxon to Euchelicerata in the ML tree, but without good support from BI or ML bootstrap analysis. Chelicerata, Euchelicerata and Arachnida as well find no good support from these inferences. So it is very likely that long-branch attraction is one major reason for the clustering of Pycnogonida and Acari in phylogenetic analysis of chelicerate relationships with mitochondrial genes, while the true position of Pycnogonida remains far from being resolved by our analyses.

### AT content and amino acid usage in chelicerate mitochondrial genomes

As mentioned above, some of the arachnid taxa show a reversal in nucleotide frequency bias (the scorpion *Centruroides limpidus*, the spiders *Ornithoctonus huwena *and *Habronattus oregonensis*, and the mite *Varroa jacobsohni*), which may be one reason for misleading results in a phylogenetic analysis [5,13]. However, it was shown before that a reversal in strand bias seems not to be the cause for the affinity between Acari and Pycnogonida [13].

With the exception of *Limulus polyphemus *all chelicerates in our phylogenetic analysis show branch lengths two or three times longer than those of hexapods, crustaceans and myriapods (Fig. 3, Fig. 4). This implies that arachnid and pycnogonid species have undergone more change in nucleotide sequence than *Limulus polyphemus *and the remainder of arthropod taxa in our study. With the exception of the scorpion *Centruroides limpidus *all arachnid and pycnogonid species in our study show a higher AT content in protein-coding genes than *Limulus polyphemus *and the outgroup taxa (Tab. 3). This is more striking if only third codon positions are compared. Strong variation in AT content between species may also lead to perturbance of phylogenetic analyses [31]. Independent evolution of higher AT content my lead to homoplastic similarities.

Looking at the amino acid composition of the protein-coding genes from chelicerates and other arthropods (Tab. 3), again strong differences are observed between *Limulus polyphemus *and the remainder of Chelicerata. Compared to mitochondrial proteins from *Limulus polyphemus *some amino acids are significantly less used in the pycnogonid species and most of the arachnids (Ala, Arg, Cys, Gln, Thr, Trp), while others are significantly more frequently used (Lys, Met). With a few exceptions, less used amino acids are coded by GC rich codons (Ala, Arg, Gly, Pro), while those more often used are coded by AT rich codons (Ile, Lys, Met, Phe, Asn). A similar effect is seen in a comparison of codon usage for the amino acids leucine and serine (Tab. 4). For the coding of leucine, UUR-codons are more frequently used in all chelicerate taxa than CUN-codons, but the difference is by far higher in those taxa, which show the highest AT-contents (The acarid taxa *Haemaphysalis flava*, *Amblyomma triguttatum*, *Varroa destructor*, *Rhipicephalus sanguineus*, and the pycnogonid *Nymphon gracile*). In contrast AGN-codons and UCN-codons for serine show only moderate variance between the taxa.

Thus the noticed derivations in amino acid usage seem to be directly linked to AT-content: the higher the proportion of adenine and thymine, the stronger the differences in amino acid usage. And in fact, for some amino acids *Nymphon gracile *together with some Acari (the taxa showing the highest AT contents as mentioned above) show the strongest differences to *Limulus polyphemus *(Ala, Gly, Phe, Trp). This fact may have further promoted the clustering of Acari and *Nymphon gracile *in our first phylogenetic analysis (Fig. 3) and in [13]. In contrast, a web spider (*Habronattus oregonensis*) which also shows high AT content does not cluster with Acari and *Nymphon gracile*, probably due to a balancing effect of the other two web spider species, which show a comparably moderate AT content.

## Conclusion

Ten individually translocated tRNA genes, a large inversion of a segment covering three protein-coding genes and five tRNA genes, and translocation of the control region lead to a derived gene order in *Nymphon gracile *compared to other arthropods, including another species of Pycnogonida (*Endeis spinosa*). If sequence data from more pycnogonid species becomes available, gene translocations may serve as phylogenetic markers, which probably resolve relationships between pycnogonid subtaxa. Phylogenetic analysis of chelicerate relationships using mitochondrial protein-coding genes supported a clade consisting of Pycnogonida and Acari. These results contradict other analyses performed with nuclear genes or morphological characters. Omiting some of the long-branching acarid species from the analysis led to a tree with unresolved relationships between Myriapoda, Pycnogonida, Xiphosura and two arachnid clades. We hypothesize that phylogenetic analyses of chelicerate interrelationships based on mitochondrial protein-coding genes is biased by three misleading factors: (a) long-branch attraction, (b) derived AT bias in all chelicerate taxa except *Limulus polyphemus*, and (c) reversed strand bias in Scorpiones, two species of Araneae and the mite *Varroa destructor*. Thus from a mitogenomic point of view the exact phylogenetic position of Pycnogonida remains an open question, but a sister group relationship between Acari and Pycnogonida as suggested by Hassanin [13] is rather caused by long-branch attraction and higher AT content than to an underlying phylogenetic signal.

## Methods

### Samples and DNA extraction

Specimens of *Nymphon gracile *were sampled at Concarneau (France) and immediately preserved in pure ethanol (99.8%). DNA from the legs of a single specimen was extracted using the DNeasy Kit (Qiagen, Germany) following the manufacturers protocol.

### PCR, sequencing and gene annotation

Fragments of six mitochondrial genes (*cox1*, *nad4*, *nad5*, *rrnl*, *rrns*) were PCR amplified using primers especially designed for this purpose. Primer sequences were as follows: cox1f: 5'-ACTAATCACA ARGAYATTGG-3'/cox1r: 5'-TAGTCTGAGT ANCGTCGWGG-3' (annealing temperature: 45°C); nad4f: 5'-TTGAGGTTAY CAGCCYG-3'/nad4r: 5'-ATATGAGCYA CAGAAGARTA AGC-3' (45°C); nad5f: 5'-AGAATTCACT AGGDTGRGAT GG-3'/nad5r: 5'-AAAGAGCCTT AAATAAAGCA TG-3' (45°C); 16Sf: 5'-GCGACCTCGA TGTTGGATTA A-3'/16Sr: 5'-CCGGTCTGAA CTCAYATC-3' (48°C); 12Sf: 5'-CAGCAKYCGC GGTTAKAC-3'/12Sr: 5'-ACACCTACTW TGTTACGACT TATCTC-3' (52°C). Primer design was performed on conserved regions of alignments using mitochondrial genes of various arthropod species [32]. All PCR experiments were done on Mastercycler and Mastercycler gradient (Eppendorf, Hamburg, Germany) using the HotMasterTaq Kit (Eppendorf). PCR reaction volumes were 50 μl (42 μl sterilized destilled water, 5 μl 10× reaction buffer 1μl dNTP mix, 1 μl primer mix (10 μM each), 1 μl DNA template, 0,2 μl = 1 u HotMasterTaq polymerase). Cycling protocol includes an initial denaturation step for 2 min at 94°C, 40 cycles of 30 sec at 94°C, 1 min at the appropriate annealing temperature (see above) and 90 sec at 68°C; in the end a final extension step for 1 min at 68°C. PCR products were gel purified (Qiaquick Gel purification kit, Qiagen, Hilden, Germany) and subsequently used for sequencing. Sequencing reactions were done using the DCTS quick start kit (Beckman-Coulter) and the CEQ 8000 capillary sequencer (Beckman-Coulter). Sequence information from these PCR fragments was used to design PCR primer pairs to amplify missing parts between them. The PCR protocol described above was therefore modified with extension steps of 7 min, and a final extension step of 3 min.

Successful amplification of PCR products was obtained with the following primer pairs (annealing temperature and approximate length in brackets): Ng-12sr: 5'-AAAAAGAATA CTAGGGTCTC TAATC C-3'/Ng-cox1f: 5'- AGCGGGTTTT ACTAATTGGT ATCC-3' (54°C, 2000 bp); Ng-cox1r: 5'- AAGAAGTTAC TAACAATATT AAAGCAGGAG G-3'/Ng-nad5f: 5'-TTTAACTATA TTCTTAGCTA GAGTATGTGC TTC-3' (58°C, 5000 bp); Ng-nad5r: 5'-ATAAAACATA AACCCCAGCA G-3'/Ng-nad4f: 5'-GATTATAGGT TGAGGAAAAT CTC-3' (49°C, 1700 bp); Ng-16sr: 5'-CGGTCTGAAC TCAGATCATG TAA-3'/Ng-12sf: 5'-TTAAAGGATA AGATGGGCTA C-3' (48°C, 1500 bp). In addition for amplification of the part spanning from *nad4 *to *rrnl *a primer pair already published in [33] was used successfully used: N4: 5'-GGAGCTTCAA CATGAGCTTT-3'/16S2: 5'-GCGACCTCGA TGTTGGATTA A-3' (50°C, 3900 bp). Sequencing of PCR products larger than 1000 bp was done using a primer walking strategy.

Primary analysis of nucleotide sequences was done using the Beckman CEQ 8000 software. Sequences were then aligned and assembled using Bioedit [34]. Protein-coding genes and ribosomal RNA genes were identified by blasting on NCBI entrez databases and by comparison with other arthropod mitochondrial genomes. Transfer RNA genes were identified using tRNAscan-SE 1.21 [19] and DOGMA [35].

### Phylogenetic analysis

Phylogenetic analysis was performed using nucleotide sequences from mitchondrial protein-coding genes. Amino acid sequences from single genes were aligned by Clustal X [36] with default settings. After retranslation to nucleotides, ambigously aligned parts were omitted from the analysis by making use of Gblocks 0.91b [37], using the "codons" option and default block parameters. Due to the results of a saturation analysis [38] on single codon positions, implemented in DAMBE, version 4.2.13 [39], third codon positions were eliminated from the alignment. The final alignment consisted of 5,711 bp for 20 taxa. A second alignment was obtained by the same procedure but omitting the two species of Acari with longest branches (*Varroa destructor *and *Leptotrombidium pallidum*), leading to an alignment consisting of 5,996 bp for 18 taxa.

Two different analyses were performed on both alignments. (1) A maximum likelihood tree was computed using PAUP* ver. 4.0b10 [40]. The model (GTR+I+gamma) and model parameters were chosen according to the AIC with modeltest 3.7 [41]. In addition 100 bootstrap replicates were performed. (2) Bayesian analysis was performed with MrBayes 3.1.2 [42]. 1,000,000 generations were run under the GTR model, with gamma distribution and a proportion of invariant sites. The first 100 out of 1000 trees were discarded as burn-in. Mitochondrial genome data from other species than *Nymphon gracile *was obtained from the OGRE database [43]. NCBI GenBank accession numbers: *Squilla mantis*, [Genbank NC_006081]; *Triops cancriformis*, [Genbank:NC_004465]; *Tricholepidion gertschi*, [Genbank:NC_005437]; *Locusta migratoria*, [Genbank:NC_001712]; *Narceus annularius*, [Genbank:NC_003343]; *Lithobius forficatus*, [Genbank:NC_002629]; *Limulus polyphemus*, [Genbank:NC_003057]; *Centruroides limpidus*, [Genbank:NC_006896]; *Heptathela hangzhouensis*, [Genbank:NC_005924]; *Ornithoctonus huwena*, [Genbank:NC_005925]; *Habronattus oregonensis*, [Genbank:NC_005942]; *Leptotrombidium pallidum*, [Genbank:NC_007601]; *Varroa destructor*, [Genbank:NC_004454]; *Ornithodoros moubata*, [Genbank:NC_004357]; *Carios capensis*, [Genbank:NC_005291]; *Ixodes hexagonus*, [Genbank:NC_002010]; *Haemaphysalis flava*, [Genbank:NC_005292]; *Rhipicephalus sanguineus*, [Genbank:NC_002074]; *Amblyomma triguttatum*, [Genbank:NC_005963].

## Abbreviations

Mitochondrial genes: *atp6*, *atp8*, ATP synthase subunits 6 and 8; *cob*, cytochrome oxidase b; *cox1-3*, cytochrome oxidase subunit I-III; *nad1-6*, *nad4L*, NADH dehydrogenase subunits 1–6, 4L; *rrns*, *rrnl*, small (12S) and large (16S) subunit ribosomal RNA; transfer RNA (tRNA) genes are listed as *trnX*, where X is replaced by the one letter amino acid code of the corresponding amino acid; CR, mitochondrial control region. EF-1α/EF-2, elongation factor-1α/-2; RNA-Pol II, RNA polymerase II. BI, Bayesian inference; ML, maximum likelihood; bp, base pairs.

## Authors' contributions

LP was primarily responsible for design and coordination of the study and conducted all the laboratory work. Analyses and manuscript draft was done in equal parts by both authors.

## Figures and Tables

**Table 1:** Annotation of the mitochondrial genome of *Nymphon gracile*

**Gene**	**Strand**	**Position number**	**Size(bp)**	**Size (aa)**	**Start codon**	**Stop codon**	**Intergenic nucleotides**
*trnY*	-	1–67	67	-	-	-	3
*trnF*	-	71–134	64	-	-	-	0
*nad5*	-	135–1809	1675	558	ATA	T	4
*trnH*	-	1814–1876	63	-	-	-	2
*nad4*	-	1879–3261	1383	460	ATG	TAA	-7
*nad4L*	-	3255–3535	281	93	ATG	TA	13
*trnT*	+	3549–3609	61	-	-	-	0
*nad6*	+	3610–4045	436	145	ATA	T	12
*cob*	+	4058–5180	1123	374	ATT	T	0
*trnS2*	+	5181–5244	64	-	-	-	8
*nad1*	-	5253–6167	915	304	ATG	TAA	0
*trnL2*	-	6169–6230	63	-	-	-	7
*trnL1*	-	6238–6299	62	-	-	-	*
*rrnL*	-	6300–7495	1196	-	-	-	*
*trnN*	-	7496–7556	61	-	-	-	6
*trnQ*	-	7563–7629	66	-	-	-	*
*rrnS*	-	7630–8396	768	-	-	-	*
*trnD*	-	8397–8460	64	-	-	-	13
*trnK*	-	8474–8540	67	-	-	-	8
*trnG*	-	8549–8611	63	-	-	-	0
*trnP*	-	8612–8674	63	-	-	-	4
*trnM*	-	8679–8743	65	-	-	-	9
*trnV*	+	8753–8820	68	-	-	-	2
*trnA*	-	8823–8882	60	-	-	-	11
*trnS1*	+	8894–8952	59	-	-	-	30
*cox2*	-	8983–9667	685	226	ATG	T	31
*cox1*	-	9699–11237	1539	512	ATA	TAA	15
*trnC*	+	11253–11314	62	-	-	-	1
*trnW*	-	11316–11385	70	-	-	-	-2
*nad2*	-	11394–12367	984	327	ATG	TAA	1
*trnI*	-	12369–12432	64	-	-	-	15
*atp8*	+	12448–12598	159	52	ATT	TAA	-7
*atp6*	+	12600–13214	615	204	ATG	TAA	1
*cox3*	+	13216–14004	789	262	ATA	TAA	17
*nad3*	+	14022–14367	346	115	ATC	T	0
*trnE*	+	14368–14434	67	-	-	-	0
*trnR*	+	14435–14490	56	-	-	-	192
CR		14491–14681	192	-	-	-	-

*Gene boundaries of rRNA genes determined by sequence of adjacent genes.

**Table 2:** Nucleotide composition and skews of *Nymphon gracile *mitochondrial protein-coding and ribosomal RNA genes.

Gene (+/- strand)	Proportion of nucleotides				%AT	AT skew	CG skew
	A	C	G	T			
atp6 (+)	0.382	0.145	0.073	0.400	78.2	-0.023	0.328
atp8 (+)	0.510	0.116	0.000	0.374	88.4	0.154	1.000
cox1 (-)	0.263	0.123	0.169	0.446	70.8	**-0.259**	**-0.158**
cox2 (-)	0.308	0.123	0.143	0.426	73.4	**-0.161**	**-0.077**
cox3 (+)	0.373	0.150	0.120	0.357	73.0	0.021	0.108
cob (+)	0.355	0.144	0.107	0.394	74.9	-0.051	0.149
nad1 (-)	0.256	0.079	0.163	0.503	75.8	**-0.326**	**-0.348**
nad2 (-)	0.318	0.070	0.116	0.496	81.4	**-0.218**	**-0.246**
nad3 (+)	0.387	0.139	0.067	0.408	79.5	-0.025	0.352
nad4 (-)	0.256	0.075	0.136	0.533	78.9	**-0.351**	**-0.288**
nad4L (-)	0.278	0.053	0.178	0.491	76.9	**-0.278**	**-0.538**
nad5 (-)	0.247	0.080	0.133	0.540	78.7	**-0.372**	**-0.249**
nad6 (+)	0.371	0.153	0.048	0.428	80.0	-0.071	0.523
rrnl(-)	0.347	0.074	0.134	0.446	79.3	**-0.125**	**-0.288**
rrns (-)	0.350	0.078	0.167	0.405	75.5	**-0.073**	**-0.363**
total (+)	0.454	0.141	0.085	0.319	77.3	0.175	0.248

AT skew ((A%-T%)/(A%+T%)) and CG skew ((C%-G%)/(C%+G%)) according to [44]. Values from (-)strand genes in bold letters.

**Table 3:** Amino acid usage and AT content of mitochondrial protein-coding genes from various arthropods.

**Taxon**	**Species**	**Ala**	**Arg**	**Asn**	**Asp**	**Cys**	**Gln**	**Glu**	**Gly**	**His**	**Ile**	**Leu**	**Lys**	**Met**	**Phe**	**Pro**	**Ser**	**Thr**	**Trp**	**Tyr**	**Val**	**AT%**	**AT%**
		GCN	CGN	AAY	GAY	TGY	CAR	GAR	GGN	CAY	ATY	TTR	AAR	ATR	TTY	CCN	TCN	ACN	TGR	TAY	GTN	PCG	PCG
												CTN					AGN					total	3rd pos.
Acari	*Ornithodoros mou*.	**123**	51	163	57	**33**	66	87	207	71	386	511	**115**	**288**	368	131	318	186	99	130	209	71,30%	78,50%
"	*Rhipicephalus san*.	**117**	**44**	**206**	58	**34**	56	79	**163**	65	**462**	487	**136**	**318**	394	120	355	142	**80**	129	139	77,90%	**89,50%**
"	*Varroa destructor*	**100**	48	**210**	54	**29**	**39**	90	**160**	65	412	507	**118**	**350**	367	**113**	317	**123**	**82**	**189**	163	79,20%	**91,70%**
"	*Amblyomma trig*.	**103**	**41**	**200**	52	**31**	51	83	**161**	63	**494**	469	**141**	**311**	**436**	**106**	352	154	**80**	130	130	78,30%	88,40%
"	*Haemaphysalis fl*.	**109**	**42**	**190**	52	**33**	**48**	81	**163**	69	**488**	476	**137**	**328**	389	**113**	362	140	**82**	135	147	76,60%	85,50%
"	*Carios capensis*	**119**	50	158	63	**32**	59	81	207	69	414	536	**120**	**293**	364	139	339	150	96	131	172	72,50%	79,90%
"	*Ixodes hexagonus*	**115**	48	161	64	**32**	**49**	79	181	67	**436**	495	**118**	**296**	385	137	385	140	95	121	182	71,10%	75,70%
"	*Leptotrombidium p*.	137	**39**	**100**	62	**26**	65	100	206	69	360	503	**147**	194	**414**	131	432	**131**	88	73	151	71,40%	83,10%
																							
Araneae	*Habronattus oreg*.	138	52	152	67	**24**	**48**	89	206	67	362	494	84	**338**	317	116	386	134	95	**158**	234	73,80%	86,10%
"	*Ornithoctonus huw*.	147	53	**109**	**74**	**23**	**48**	96	213	73	358	469	97	**273**	355	134	402	139	99	127	260	69,70%	78,40%
"	*Heptathela han*.	135	53	136	54	**29**	64	90	218	73	369	561	100	**274**	344	140	375	151	100	114	192	71,40%	82,10%
																							
Scorpiones	*Centruroides lim*.	190	63	**93**	65	43	**46**	86	253	77	**250**	604	79	184	355	145	376	176	103	120	**298**	62,90%	68,10%
																							
Pycnogonida	*Nymphon gracile*	**125**	53	160	69	**35**	**46**	80	188	71	362	494	**123**	**286**	**420**	136	370	158	**82**	139	218	76,60%	**90,50%**
																							
Xiphosura	*Limulus polyph*.	176	62	147	59	49	67	86	237	78	346	565	83	212	330	152	387	178	110	121	221	66,30%	74,70%
																							
Myriapoda	*Narceus ann*.	205	50	110	66	**31**	80	74	276	75	298	606	68	196	276	162	342	206	113	128	**283**	62,10%	67,90%
"	*Lithobius for*.	193	60	125	70	**34**	68	76	256	74	331	557	85	246	306	137	357	199	102	140	239	65,70%	72,00%
																							
Crustacea	*Squilla mantis*	**225**	62	132	**74**	42	73	82	259	80	272	560	83	235	317	136	324	210	97	**152**	243	68,10%	79,30%
"	*Triops can*.	207	61	164	69	**32**	79	78	232	81	316	627	73	199	319	138	351	188	107	137	220	68,20%	77,70%
																							
Hexapoda	*Tricholepidion ger*.	184	58	148	72	44	73	76	245	80	324	564	74	234	310	144	376	223	104	147	226	67,60%	76,90%

Bold numbers indicate strong differences (+/-25%) to *Limulus polyphemus *(underlined). This species was chosen for comparison, because in the phylogenetic analyses (Fig. 3, 4) it shows a branch length comparable to those of crustacean, myriapod and hexapod species, while the remainder of chelicerates was subject to a higher degree of nucleotide substitution. See methods for complete names of species.

**Table 4:** Codon usage for leucine and serine codons of chelicerate arthropods. See Fig. 3 for full species names.

**Codon**	**Amino acid**	**Xiph.**	**Pycn.**	**Scor.**	**Araneae**			**Acari**							
		*L.pol.*	*N.g.*	*C.l.*	*H.h.*	*O.h.*	*H.o.*	*L.pal.*	*I.h.*	*C.c.*	*H.f.*	*A.t.*	*V.d.*	*R.s.*	*O.m.*
UUA	L	226	363	154	321	212	301	245	265	314	314	328	380	355	242
UUG	L	84	51	163	49	118	70	44	52	56	45	28	29	24	84
**UUR**	L	**310**	**414**	**317**	**370**	**330**	**371**	**289**	**317**	**370**	**359**	**356**	**409**	**379**	**326**
															
CUA	L	94	27	87	81	62	81	59	70	73	50	33	40	42	70
CUC	L	55	1	62	16	13	8	28	37	30	9	10	6	6	16
CUG	L	4	2	28	16	7	4	7	7	2	7	1	1	1	5
CUU	L	106	43	110	78	57	31	120	67	64	52	70	58	59	95
**CUN**	L	**259**	**73**	**287**	**191**	**139**	**124**	**214**	**181**	**169**	**118**	**114**	**105**	**108**	**186**
															
AGA	S	66	67	40	83	99	91	89	64	63	74	60	84	72	71
AGC	S	8	1	28	8	5	6	2	2	12	5	3	6	2	8
AGG	S	12	18	17	1	12	7	7	15	2	7	6	2	7	10
AGU	S	22	45	44	22	16	30	29	32	30	26	22	61	30	29
**AGN**	S	**108**	**131**	**129**	**114**	**132**	**134**	**127**	**113**	**107**	**112**	**91**	**153**	**111**	**118**
															
UCA	S	98	60	43	85	97	95	101	99	100	119	127	39	129	82
UCC	S	65	8	47	49	40	31	49	58	46	26	28	16	25	23
UCG	S	6	7	12	7	15	8	14	6	4	8	8	4	1	8
UCU	S	110	163	145	121	119	118	141	110	86	98	98	108	89	89
**UCN**	S	**279**	**238**	**247**	**262**	**271**	**252**	**305**	**273**	**236**	**251**	**261**	**167**	**244**	**202**
